# Factors influencing nurses’ behavioral intention toward caring for COVID-19 patients on mechanical ventilation: A cross-sectional study

**DOI:** 10.1371/journal.pone.0259658

**Published:** 2021-11-05

**Authors:** Jingxia Cheng, Jinbo Cui, Wenwen Yu, Hua Kang, Yongming Tian, Xiaolian Jiang

**Affiliations:** 1 West China School of Nursing, Sichuan University/West China Hospital, Sichuan University, Sichuan, China; 2 College of Nursing, Chengdu University of Traditional Chinese Medicine, Sichuan, China; 3 West China Hospital, Sichuan University, Sichuan, China; Sunway University, MALAYSIA

## Abstract

**Objectives:**

To investigate nurses’ behavioral intention toward caring for COVID-19 patients on mechanical ventilation, as well as the factors affecting their intention.

**Background:**

COVID-19 patients undergoing mechanical ventilation have many care needs and pose more challenges for nurses, which might adversely affect nurses’ intention toward caring behavior.

**Methods:**

A cross‐sectional study was conducted by using simple random sampling to recruit 598 nurses from five tertiary hospitals in Sichuan Province, China. The participants responded to an online questionnaire that included questions on demographic characteristics; the Attitude, Subjective Norms, and Behavioral Intention of Nurses toward Mechanically Ventilated Patients (ASIMP) questionnaire; the Nursing Professional Identity Scale (NPIS); and the Compassion Fatigue-Short Scale (CF-Short Scale). ANOVA, Spearman correlation analysis, and multiple linear regression were performed to analyze the data.

**Results:**

The mean total behavioral intention score was 179.46 (± 14.83) out of a total score of 189.00, which represented a high level of intention toward caring for patients on mechanical ventilation. Multiple linear regression revealed that subjective norms (β = 0.390, *P*<0.001), perceived behavioral control (β = 0.149, *P*<0.001), professional identity (β = 0.101, *P* = 0.009), and compassion fatigue (β = 0.088 *P* = 0.024) were significant predictors of nurses’ behavioral intention.

**Conclusions:**

Most nurses have a positive behavioral intention to care for COVID-19 patients undergoing mechanical ventilation. The findings in this study provide some insight for developing effective and tailored strategies to promote nurses’ behavioral intention toward caring for ventilated patients under the pandemic situation.

## Background

The outbreak of COVID-19 was declared a pandemic by the WHO on 11^th^ March 2020 [1]. The novel strain of coronavirus has caused a more challenging pneumonia pandemic than previous epidemics due to the high contagiousness, substantial variability, low level of knowledge regarding the course of infection, and lack of established treatments or vaccines in the early stages. At the time of writing (October 2021), COVID-19 had accounted for 233,503,524 confirmed cases and 4,777,503 deaths [2] and had had enormous impacts on many aspects, from health to sociopolitical and economic concerns, on a global scale.

As the global response to COVID-19 continues, healthcare systems have encountered operations exceeding capacity, a lack of sufficient protective equipment, an overstretched nursing workforce, and excessive workload [3, 4]. Regarding the burden of COVID-19 on health care resources, it has been found that approximately 5–32% of confirmed cases required admission to the intensive care unit (ICU) [5–8], with more than two-thirds of those requiring invasive mechanical ventilation due to COVID-19-related shortness of breath resulting in hypoxemic respiratory failure [9].

Mechanical ventilation is necessary for critical patients, but it is uncomfortable and irritating, which increases the difficulties and burdens of care [10]. In addition, mechanical ventilation places health care workers at a higher risk of transmission of COVID-19 [11]. Greater challenges have been posed for nurses’ work when caring for COVID-19 patients on mechanical ventilation, which relates to professional skill and ethics in nursing. Earlier studies have reported the problems nurses suffered during the pandemic, including loneliness, anxiety, fear, fatigue, and sleep disorders [4, 12, 13]. These terrible problems generated by COVID-19 can affect nurses’ careers and threaten the quality of care by affecting nurses’ intention to care for COVID patients [14] because of nurses’ multifactorial role in managing a patient on mechanical ventilation. Thus, it is of great importance to examine nurses’ behavioral intention to care for these patients during the COVID-19 pandemic and the factors affecting their intention.

"Behavioral intentions are instructions that people give to themselves to behave in certain ways or people’s decisions to perform particular actions," which can be concluded from one’s responses of "I will do something" or "I intend to do something" [15]. Behavioral models and theories have been used to investigate nurses’ intention to participate in a crisis, and the most commonly used theory for studying the factors that influence nurses’ behavioral intention is Ajzen’s theory of planned behavior (TPB) [16]. According to the TPB, one’s behavioral intention is determined by three direct factors: attitude toward the behavior, subjective norms, and perception of behavioral control [17]. Generally, the more positive the attitude and subjective norm and the greater the perceived control, the stronger the person’s intention to perform the behavior [17]. The variables in the TPB, including attitude, subjective norms, and perceived behavior control, can be measured directly by asking questions or indirectly based on beliefs. Compared to the direct approach, belief-based measures have the advantage of providing insight into the cognitive foundation underlying perceptions of behavioral control [16]. Researchers have been more inclined to combine direct and indirect approaches to develop instruments. In indirect assessments, each subscale is examined with two subsets: the behavioral belief and outcome evaluation subset, the normative belief and motivation-to-comply subset, and the control belief and perceived power subset [18].

The TPB has been found to explain 20–30% of nurses’ intention toward caring for patients with emerging infectious diseases [19]. Thus, as a norm identifying behaviors that all nurses are expected to follow, nursing professional values and moral norms were added into the theoretical framework to enhance the predictive power of the TPB [19, 20]. However, although nursing professional values added to the explained variance in nursing intention beyond the TPB, there has been no consistent conclusion that professional value is a significant predictor of nurses’ behavioral intention [21]. Professional identity, which is usually defined as including both personal and professional development and involves the internalization of values and perspectives, includes broader professionalism. Further exploration of the relationship between professional identity and nurses’ behavioral intention is necessary and valuable.

Compassion fatigue consists of two parts: burnout, which refers to exhaustion, frustration, anger, and depression, and secondary traumatic stress, which is a negative feeling driven by fear and work-related trauma [22]. Since the COVID-19 pandemic, nurses, as the largest group of healthcare workers, nurses contacted patients firstly, directly, and continuously and they have suffered from many stressors, such as over workload, psychological stress, and ethical challenges [4, 23]. Compassion fatigue has been found to be a predictor of caring behaviors [22] and care quality [24]. Nevertheless, there has been a lack of studies attempting to investigate the relationship between compassion fatigue and nurses’ behavioral intention toward caring for patients.

A recent study investigated the intentional behavior of hospital staff to care for COVID-19 patients, concluding that healthcare staff who cared for COVID-19 patients had high behavioral intention to continue caring for them [25]. Subjective norms and participation in care for COVID-19 patients were predictors of behavioral intention. However, there is limited evidence regarding nurses’ intention to care for COVID-19 patients on mechanical ventilation, which involves a higher burden and greater risk of infection. Thus, this study was conducted to investigate nurses’ behavioral intention toward caring for COVID-19 patients on mechanical ventilation and the factors affecting their intention.

## Methods

### Study design, setting and participants

**Fig 1:**
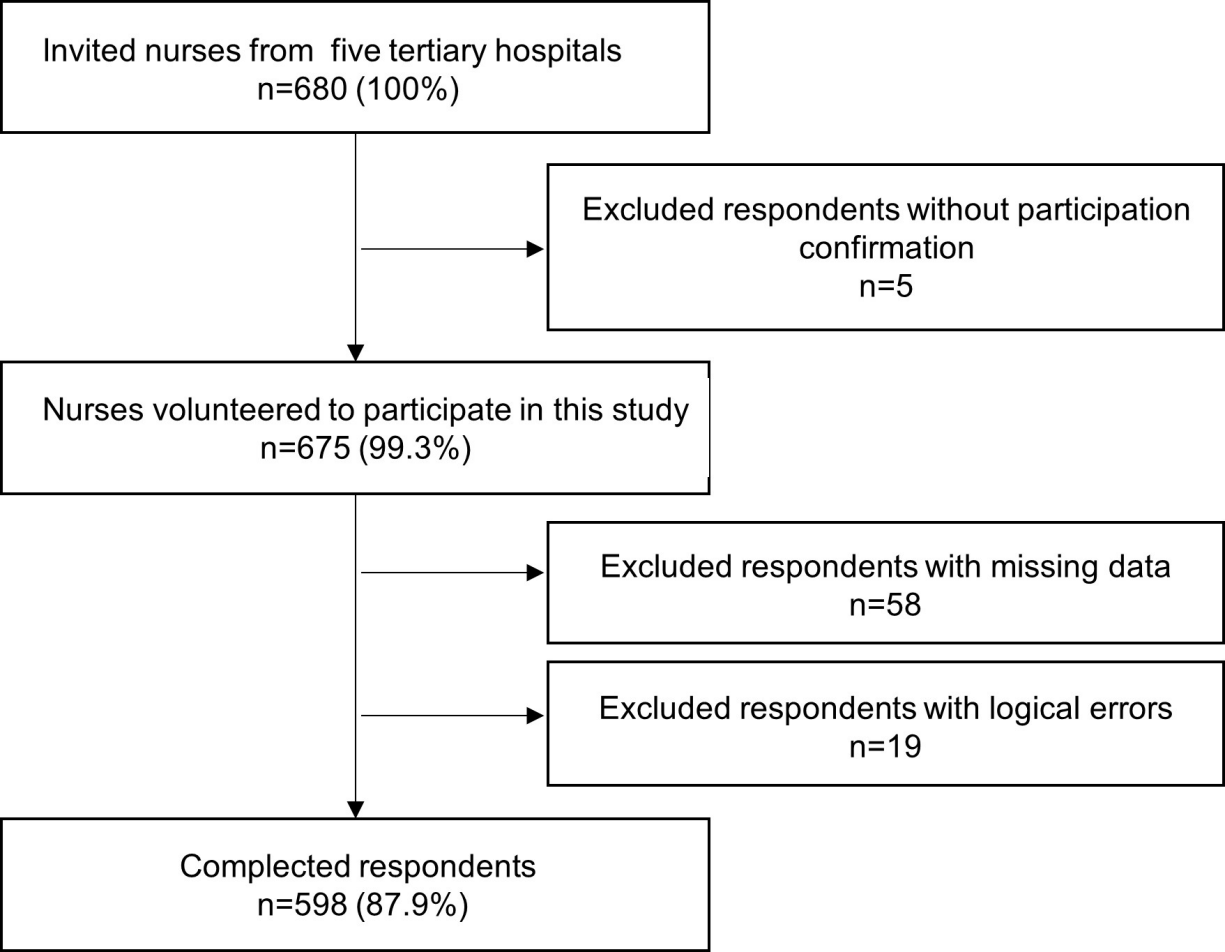
Recruitment procedure.

A cross-sectional study was conducted from November 2020 to December 2020. A list of all nurses working at the respiratory departments and ICUs of five tertiary hospitals in Sichuan Province, China, was obtained; the nurses on the list were numbered, and then simple random sampling was performed through the sampling function of Excel by computer. The sample size was calculated as 25% of all 2200 nurses working in the respiratory departments and ICUs of the five tertiary hospitals [26]. Based on a nonresponse rate of 20%, 680 nurses were invited to participate. Valid responses were received from a total of 598 nurses after respondents without participation confirmation or with logical errors were excluded, for a response rate of 87.9%. The recruitment procedure is shown in Fig 1.

All participants reviewed the explanation of the study and agreed to voluntarily participate by checking the confirmation box on the first page of the questionnaire; they were informed that participation was voluntary, that their personal and institutional information would be kept confidential, and that they could withdraw at any time during the survey. Ethical clearance for the study was obtained from the Biomedical Ethics Committee of the relevant hospital (No. 2020 (981)).

### Instruments

The self-administered questionnaire consisted of four parts:

Part A: The demographic and occupational characteristics collected from participants included gender, age, education level, years of nursing experience, years of nursing experience in the ICU or respiratory ward, department, professional title, completion of training about caring for patients on mechanical ventilation, completion of training about caring for patients with infectious diseases, experience caring for mechanically ventilated patients with infectious diseases other than COVID-19, experience working on the front-line of COVID-19 anti-pandemic work, and experience caring for mechanically ventilated patients with COVID-19.

Part B: The Attitude, Subjective Norms, and Behavioral Intention of Nurses toward Mechanically Ventilated Patients (ASIMP) questionnaire developed by Meyrick Chum-Ming CHOW [27] and revised by Kang Hua [28] was used in this study. This study added the measurements of perceived behavioral control based on TPB and behavioral intention toward occupational protection to make the scale more applicable for the pandemic situation. New items were confirmed by consulting the literature and validated through expert review. The content validity indexes of each part of the ASPIMP were between 0.79 and 1.00. The Cronbach’s α coefficients were between 0.62 and 0.92. The scale consisted of 86 items: (a) attitude: direct questions (3 items), behavioral beliefs (13 items), behavioral outcome evaluation (13 items); (b) subjective norms: direct question (1 item), normative beliefs (4 items), motivation to comply (4 items); (c) perceived behavioral control: direct question (1 item), control beliefs (10 items), perceived power (10 items); (d) behavioral intention (27 items). The questionnaire used a 7-point Likert scale ranging from 1 (strongly disagree) to 7 (strongly agree). Negative statements were coded in reverse. The subscale scores, including attitude, subjective norms, and perceived behavioral control, were scored by the formula: $\sum_{i=1}^{p}a_{i}*b_{i}+c$ (*a_i_*, *b_i_* means the score of corresponding items of the two subsets in each subscale, *p* means the number of items of each subset, *c* means the total score of direct questions).

Part C: The Nursing Professional Identity Scale (NPIS) developed by the Department of Medicine at the University of Tokyo and revised by Zhao Hong [29] was adopted to assess the participants’ professional identity. The content validity index was greater than 0.80, and the Cronbach’s α coefficients were between 0.69 and 0.84 [29]. The scale contained 21 items rated on a 7-point Likert scale, and a higher score indicated a higher level of professional identity.

Part D: The Compassion Fatigue-Short Scale (CF-Short Scale) was compiled by Adam in 2006 and revised by Lou Baona [30]. The Cronbach’s α coefficients were 0.87 for the total scale, 0.79 for the dimension of secondary trauma, and 0.85 for the dimension of job burnout [30]. The scale consists of secondary trauma and job burnout subscales, including 5 and 8 items, respectively, with a total of 13 items rated on a 10-point Likert scale from 1 (never) to 10 (very frequent). The higher the total score was, the more severe the compassion fatigue.

### Data collection

Data collection was conducted by using an online questionnaire. We uploaded the questionnaire to Wenjuanxing (https://www.wjx.cn), an online questionnaire system widely used in China, to create a questionnaire link. Then, the questionnaire link was sent to nurses recruited in this study through WeChat or QQ. To prevent duplicate responses, we accepted only one questionnaire per person by tracking internet protocol addresses. Two research assistants were responsible for checking and distributing the questionnaire.

### Data analysis

Frequency distributions were used to describe demographic and occupational characteristics. Scores of the ASPIMP, NPIS, and CF-Short Scale were described by means with SDs after testing for skewness and kurtosis. One-way ANOVA was used to test for a difference in mean values between two or more groups. Spearman correlations and multiple linear regression were performed to examine the predictive power of attitude, subjective norms, perceived behavioral control, professional identity, and compassion fatigue on nurses’ behavioral intention to care for COVID-19 patients on mechanical ventilation. Data were analyzed with SPSS 23.0. Statistical significance was set at p < 0.05 (two-tailed).

## Results

### Demographic and occupational characteristics of participants

More than ninety percent (93.1%, n = 557) of participants were female, and two-thirds of participants (69.2%, n = 414) were between 25~35 years old. Most of them had an undergraduate or graduate degree (71.7%, n = 429) and junior professional title (nurse or senior nurse) (77.6%, n = 464) and had worked in nursing for less than 10 years (68.4%, n = 409). Over 80% of participants had received training on mechanical ventilation (84.3%, n = 504) or caring for patients with infectious disease (92.5%, n = 553), and half of them (58.4%, n = 349) had experience caring for mechanically ventilated patients with infectious diseases. However, only one-fourth (25.3%, n = 151) of survey respondents had participated in the front-line work for COVID-19 prevention and control, and 15.1% (n = 90) had experience caring for mechanically ventilated COVID-19 patients (Table 1).

**Table 1:** Demographic and occupational characteristics of participants and differences in behavioral intention toward caring for COVID-19 patients on mechanical ventilation.

Categories	N (%) (n = 598)	Behavioral intention			
		Mean	SD	*F*	*P*
**Gender**					
Male	41 (6.9)	176.44	20.87	1.833	0.176
Female	557(93.1)	179.69	14.29		
**Age**					
<25	71(11.9)	180.47	13.09	1.258	0.286
25~30	241(40.3)	178.92	15.78		
31~35	173(28.9)	178.23	16.15		
36~40	67(11.2)	181.42	11.03		
>40	46((7.7)	182.57	11.08		
**Education level**					
Junior college or below	169 (28.3)	178.65	14.13	0.717	0.398
Undergraduate or above	429 (71.7)	179.79	15.10		
**Years of nursing experience in the ICU or respiratory ward**					
<1	277(46.3)	180.05	14.30	1.607	0.187
1~5	154(25.8)	178.22	16.52		
6~10	106(17.7)	178.07	15.79		
>10	61(10.2)	182.38	9.90		
**Years of nursing experience**					
<5	185(30.9)	180.02	13.67	5.358	0.005*
6~10	224(37.5)	177.08	17.53		
>10	189(31.6)	181.75	11.77		
**Department**					
ICU	293 (49.0)	179.44	14.40	0.01	0.970
Respiratory unit	305 (51.0)	179.49	15.26		
**Professional title**					
Junior title (nurse)	125 (20.9)	179.50	13.45	2.598	0.075**
Junior title (senior nurse)	339 (56.7)	178.48	16.30		
Intermediate title or above (supervisor nurse or above)	134 (22.4)	181.92	11.61		
**Training on caring for patients on mechanical ventilation**					
Yes	504 (84.3)	179.31	14.91	0.362	0.548
No	94 (15.7)	180.31	14.43		
**Training on caring for patients with infectious diseases**					
Yes	553 (92.5)	179.75	14.20	2.730	0.099**
No	45 (7.5)	175.96	21.03		
**Experience caring for mechanically ventilated patients with infectious diseases other than COVID-19**					
Yes	349 (58.4)	178.69	15.46	2.280	0.132
No	249 (41.6)	180.55	13.86		
**Experience working on the front-line of COVID-19 anti-pandemic work**					
Yes	151 (25.3)	178.80	16.65	0.402	0.526
No	447 (74.7)	179.69	14.18		
**Experience caring for mechanically ventilated patients with COVID-19**					
Yes	90 (15.1)	176.92	18.30	3.121	0.078**
No	508 (84.9)	179.91	14.10		

* P < 0.05.

** P < 0.1.

### Attitude, subjective norms, and perceived behavioral control of nurses in caring for COVID-19 patients on mechanical ventilation

The mean total scores of attitudes, subjective norms, and perceived behavioral control were 372.99 (± 101.53), 158.80 (± 40.53), and 212.32 (± 38.67), respectively, representing a high level of attitude, subjective norms, and perceived behavioral control (Table 2).

**Table 2:** The attitude, subjective norm, perceived behavioral control, professional identity, compassion fatigue and behavioral intention of nurse to care for COVID-19 patients on mechanical ventilation.

Variable	range	Mean	SD
Attitude	16~658	372.99	101.53
Subjective norms	5~203	158.80	40.53
Perceived behavioral control	11~497	212.32	38.67
Professional identity	21–105	83.93	13.62
Compassion fatigue	13~130	48.79	25.95
Behavioral intention	27~189	179.46	14.83

### Professional identity and compassion fatigue of nurses

The mean total scores of nurses’ professional identity and compassion fatigue were 83.93 (± 13.62) and 48.79 (± 25.95), respectively, indicating that most nurses had a high level of professional identity and a low level of compassion fatigue (Table 2).

### Intention to care for COVID-19 patients on mechanical ventilation

The mean total score of behavioral intention toward caring for COVID-19 patients on mechanical ventilation was 179.46 (± 14.83) out of a total score of 189.0, which represents a high level of intention (Table 2). Of all 27 items, behavioral intention toward “performing chest physical therapy every 4 hours”, “auscultating the breath sounds of the patient to ensure airway patency” and “placing the patient in Fowler’s position if conditions permit” had the lowest mean score (S1 Table).

### Factors influencing behavioral intention toward caring for COVID-19 patients on mechanical ventilation

The results of the univariate analysis revealed that years of nursing experience had a significant relationship with nurses’ behavioral intention (P < 0.05) (Table 1). Spearman correlation analysis indicated moderate and positive relationships among subjective norms, perceived behavioral control, professional identity, compassion fatigue, and behavioral intention (Table 3). To avoid the omission of possible important factors, the variables that theoretically recommended or that showed a univariate relationship with behavioral intention (P < 0.1), including years of nursing experience, professional title, completion of training on infectious disease care, experience caring for mechanically ventilated patients with COVID-19, attitude, subjective norm, perceived behavioral control, professional identity, and compassion fatigue, were entered into the multiple linear regression.

**Table 3:** Correlations among attitude, subjective norms, perceived behavioral control, professional identity, compassion fatigue, and behavioral intention.

	Variable	1	2	3	4	5	6
1	Attitude	1					
2	Subjective norms	0.002	1				
3	Perceived behavioral control	-0.029	0.362**	1			
4	Professional identity	-0.008	0.395**	0.313**	1		
5	Compassion fatigue	0.061	-0.299**	-0.370**	-0.363**	1	
6	Behavioral intention	-0.019	0.490**	0.335**	0.373**	-0.332**	1

**P<0.01.

The results of multivariate regression are summarized in Table 4, which indicated that approximately 30.5% of the total variation in nurses’ behavioral intention toward caring for COVID-19 patients on mechanical ventilation could be explained by subjective norms (β = 0.39, P < 0.001), perceived behavioral control (β = 0.15, P < 0.001), professional identity (β = 0.10, P = 0.009), and compassion fatigue (β = 0.09, P = 0.024).

**Table 4:** Multiple linear regression of behavioral intention toward caring for COVID-19 patients on mechanical ventilation.

Variable	β	SD	95%CI (Lower, Upper)	*t*	*P*
Years of nursing experience	0.057	0.106	(-0.151,0.265)	1.177	0.240
Professional title	0.024	1.094	(-2.120,2.168)	-0.490	0.624
Training on caring for patients with infectious diseases	0.021	1.963	(-3.826,3.868)	-0.613	0.540
Experiencing caring for mechanically ventilated patients with COVID-19	0.019	1.466	(-2.854,2.892)	-0.538	0.591
Attitude	-0.060	0.005	(-0.070, 0.050)	-1.708	0.088
Subjective norms	0.390	0.014	(0.363, 0.417)	10.102	<0.001
Perceived behavioral control	0.149	0.015	(0.120, 0.178)	3.853	<0.001
Professional identity	0.101	0.042	(0.019, 0.183)	2.615	0.009
Compassion fatigue	-0.088	0.022	(-0.131, -0.045)	-2.256	0.024

R^2^ = 0.305.

## Discussion

The primary findings of this study revealed that participants’ behavioral intention toward caring for mechanically ventilated patients with COVID-19 was positive, which is similar to the results of the study conducted on behavioral intention toward caring mechanically ventilated patients under non-pandemic situations [28], as well as a study investigating the behavioral intention of hospital staff to care for COVID-19 patients [25]. The finding was encouraging and indicated that nurses are willing to take care of mechanically ventilated patients regardless of the pandemic situation. Although the pandemic has impacted their work and health, nurses’ perseverance and sense of responsibility have led them to respond positively despite the crisis [25, 31].

In this study, demographic and occupational characteristics did not explain behavioral intention in the multivariate analyses. However, this finding needs to be treated with caution because the significant difference in the sample size between groups might have affected the power of the test, and the impact of some crucial variables should not be ignored. Earlier studies conducted by Kang Hua [28] and Lee [19] indicated the impact of education level, years of nursing experience, and training programs on nurses’ behavioral intention to care for ventilated patients and patients with emerging infectious diseases, which explained that a higher level of education and greater nursing experience or training was beneficial for improving nurses’ professional knowledge, skill proficiency, self-efficacy, and capability to cope with difficulties, which made them more confident, positive, and interested in implementing caring behaviors in the context of challenges [19, 28]. The current study found that participants who provided care for COVID-19 patients had higher behavioral intention scores [25]. More studies are suggested to clarify the role of demographic and occupational characteristics regarding nurses’ behavioral intention under the pandemic situation.

This study examined factors related to nurses’ behavioral intention based on the TPB, and subjective norms and perceived behavioral control were found to be significant predictors. The findings are similar to those of the current studies on the intentional behavior of caring for COVID-19 patients [25]. In this paper, subjective norms, defined as the perceived social pressure from head nurses, peers, and doctors to engage or not engage in caring behavior, were indicated to have the strongest influence on behavioral intention. In contrast, a survey conducted on Korean nurses’ intention to care for patients with emerging infectious diseases showed that subjective norms did not explain behavioral intention, with one possible explanation being that although nurses could not refuse instructions from their superiors or nursing managers because of the hierarchical culture, they were subjectively unwilling to care for patients with emerging infectious diseases, especially when lacking relevant knowledge [19]. In our study, most nurses had positive intentions and attitudes toward caring for mechanically ventilated patients with COVID-19. Thus, although the influence of important people on nurses’ behavioral intention was significant, a positive attitude was equally important.

However, unexpectedly, attitude was found to not be significant in predicting nurses’ intention, which is inconsistent with reports from previous studies [19, 28]. One possible explanation was that nurses were portrayed by the media during the pandemic as being heroic and warm-hearted and having a strong sense of professional morality [32]. The demand for public image and the Chinese culture of unity and collectivism might have weakened the influence of personal attitudes and strengthened the influence of organizations and influential people. The results warrant further exploration.

Although weaker than the predictive power of subjective norms, perceived behavioral control was confirmed to be a predictor of behavioral intention, concurring with previous studies [19, 33, 34]. As a new variable introduced to the TPB, perceived behavioral control is a key variable to enhance the predictive power of the TPB [16]. The COVID-19 pandemic gives rise to some control factors, including scarce medical resources, shortage of protective equipment, higher occupational risks, heavier workload, independent work, and role conflicts. These factors facilitate or impede nurses’ behavioral intention by shaping their perceived behavioral control, which is the perception of those control factors’ likelihood and influential power [17]. Thus, it is imperative to promote facilitating factors and minimize impeding factors to enhance perceived behavioral control.

Using a creative design, the present study also explored the influence of professional identity and compassion fatigue on nurses’ behavioral intention because professional identity and compassion were reported to have significant influences on every aspect of nursing practice [35–37]. Meanwhile, these two factors can be affected by the COVID-19 pandemic through changes in the social and working settings [32, 38–42]. The results of multivariate analysis showed that nurses with a higher level of professional identity and lower level of compassion fatigue had more positive intentions toward caring for COVID-19 patients on mechanical ventilation. Nevertheless, there seemed to be no unified conclusion across different studies [19, 43, 44] about the influence of professional identity. A possible explanation was that researchers focused on different aspects of professionalism. Nurses’ behavior in taking care of patients with emerging infectious diseases, such as SARS, was more influenced by organizational commitment and the strength of an individual’s identification with and involvement in the goals and values of a particular organization than by their professional commitment [45]. In our study, the measurement of professional identity included seven dimensions, namely, role identity, understanding of the role, sense of professional value, self-efficacy, sense of self-determination, sense of influence on the organization and sense of influence on the patient, and these dimensions could fully reveal how nurses viewed their professions.

Compassion fatigue is a phenomenon of exhaustion and dysfunction in healthcare workers resulting from prolonged exposure to work-related stress and compassion stress [46]. Although few studies have focused on the association between behavioral intention and compassion fatigue, as a well-known phenomenon among clinical nurses, compassion fatigue has been shown to reduce productivity, increase staff turnover and sick days and lead to patient dissatisfaction and risks to patient safety [46–48]. Therefore, it is not difficult to interpret the negative influence of compassion fatigue on behavioral intention.

Nurses are already and will continue to be the first responders in the context of the global pandemic [49]. As the immediate antecedent of behavior [16], nurses’ behavioral intention and its related factors are crucial to designing tailored interventions that foster nurses’ caring behavior. This study is the first to focus on nurses’ behavioral intention toward caring for COVID-19 patients undergoing mechanical ventilation, creatively exploring the impact of professional identity and compassion fatigue. Our novel findings indicated that subjective norms, perceived behavioral control, professional identity, and compassion fatigue were significant predictors of behavioral intention. Thus, clear instructions from nursing managers, support from peers, and advice from doctors are necessary to encourage nurses to take care of COVID-19 patients on mechanical ventilation. Meanwhile, it might be worth developing strategies to enhance behavioral control and professional identity and reduce compassion fatigue to promote behavioral intention toward caring for COVID-19 patients on mechanical ventilation.

Despite the significant findings, some limitations exist. First, this study investigated behavioral intention toward caring for COVID-19 patients on mechanical ventilation rather than actual caring behavior. Only 15.1% of the participants had taken care of COVID-19 patients on mechanical ventilation. Compared with behavior, behavioral intention may be overestimated. However, our study could provide evidence for future research. Second, this study collected self-report data with inevitable bias; our large sample size and random sampling may have reduced bias.

## Conclusions

Most nurses in this study had a positive behavioral intention to care for COVID-19 patients on mechanical ventilation. Nurses with a positive intention were found to have a higher level of subjective norms, perceived behavioral control, and professional identity and a lower level of compassion fatigue, with subjective norms having the most significant predictive power. These findings provide some insight for developing effective and tailored strategies to promote nurses’ behavioral intention toward caring for ventilated patients under the pandemic situation.

## Supporting information

S1 Table The score of items of nurses’ behavioral intention toward caring for COVID-19 patients on mechanical ventilation. (DOCX)

S1 File Research data. (XLSX)
