# Supplementary material for: Factors influencing nurses’ behavioral intention toward caring for COVID-19 patients on mechanical ventilation: A cross-sectional study
Source: PLoS One. 2021 Nov 5;16(11):e0259658. doi: 10.1371/journal.pone.0259658 (PMC8570482; doi:10.1371/journal.pone.0259658)
Supplement: S1 Table — (DOCX) [file pone.0259658.s001.docx]

**S1 Table. The Score of Items of Nurses’ Behavioral Intention toward Caring for COVID-19 Patients on Mechanical Ventilation.**

| Items | | Range | | Mean | | SD | |  |  |
| --- | --- | --- | --- | --- | --- | --- | --- | --- | --- |
|  |  |  |  |  |  |  |  |  | |
| **When caring for COVID-19 patients with mechanical ventilation, I would:** | |  | |  | |  | |  | |
| Perform chest physical therapy every 4 hours (using a vibrometer or tapping the patient's back to remove sputum). | | 1~7 | | 6.26 | | 1.08 | |  | |
| Auscultate the breath sounds of the patient to ensure airway patency regularly. | | 1~7 | | 6.46 | | 1.03 | |  | |
| Place the patient in the fowler position if the condition permits. | | 1~7 | | 6.51 | | 0.83 | |  | |
| Monitor and interpret waveforms for pressure, volume, and flow in the graphic display (if available). | | 1~7 | | 6.54 | | 0.83 | |  | |
| Change the patient's position at least 2 hourly. | | 1~7 | | 6.59 | | 0.86 | |  | |
| Explain the purpose of mechanical ventilation, if the patient is conscious. | | 1~7 | | 6.59 | | 0.79 | |  | |
| Try to identify (and resolve, if appropriate) the underlying problem(s) which accounts for the restlessness of the patient. | | 1~7 | | 6.60 | | 0.70 | |  | |
| Adjust the temperature of the humidifier according to sputum properties. | | 1~7 | | 6.61 | | 0.78 | |  | |
| Paying close attention to the results of blood gas analysis. | | 1~7 | | 6.61 | | 0.75 | |  | |
| Change ventilator circuit and filters timely. (with another nurse to help ventilate the patient with a manual resuscitator) whenever necessary. | | 1~7 | | 6.61 | | 0.80 | |  | |
| Encourage and facilitate the patient to communicate with me. | | 1~7 | | 6.62 | | 0.69 | |  | |
| Set appropriate alarm limits for the patient. | | 1~7 | | 6.64 | | 0.75 | |  | |
| Apply vaseline gauze to keep an unconscious patient's eyes covered. | | 1~7 | | 6.65 | | 0.70 | |  | |
| Assess patient's nutritional status regularly. | | 1~7 | | 6.66 | | 0.69 | |  | |
| Perform suction therapy whenever necessary. | | 1~7 | | 6.67 | | 0.72 | |  | |
| Check the ventilator settings against prescription. | | 1~7 | | 6.68 | | 0.71 | |  | |
| Evaluate the cuff pressure of the endotracheal / tracheostomy tube regularly. | | 1~7 | | 6.68 | | 0.71 | |  | |
| Perform oral care for the patient. | | 1~7 | | 6.69 | | 0.64 | |  | |
| Assess the patient's condition before, during, and after weaning from the ventilator. | | 1~7 | | 6.71 | | 0.64 | |  | |
| Respond to ventilator alarm and resolve the underlying problem timely. | | 1~7 | | 6.72 | | 0.64 | |  | |
| Keep the water bottle of the ventilator in a low position and remove condensate from the pipe and water bottle in time. | | 1~7 | | 6.73 | | 0.69 | |  | |
| Take measures to prevent cross-infection in the hospital. | | 1~7 | | 6.74 | | 0.61 | |  | |
| Take measures to protect the patient and me from COVID-19. | | 1~7 | | 6.76 | | 0.59 | |  | |
| Check the position of the patient's endotracheal tube regularly. | | 1~7 | | 6.77 | | 0.59 | |  | |
| Change the distilled water in the humidifier daily and add it in time when the distilled water is rare. | | 1~7 | | 6.78 | | 0.62 | |  | |
| Keep the ventilator pipeline unobstructed and prevent excessive pulling, twisting, folding, or loosening. | | 1~7 | | 6.78 | | 0.58 | |  | |
| Monitor patient's SaO_2_ dynamically. | | 1~7 | | 6.79 | | 0.57 | |  | |
